# Refractory Chronic Plaque Psoriasis and Psoriatic Arthritis Successfully Managed With Combined Secukinumab and Apremilast Therapy: A Case Report

**DOI:** 10.7759/cureus.96555

**Published:** 2025-11-11

**Authors:** Reem AlQusaimi, Fawziah AlRujaib, Fahad AlSharhan, Doaa AlAwadhi, Suad Alassaf, Fatemah Abdulrahman

**Affiliations:** 1 Dermatology, Abdulkareem AlSaeed Dermatology Center, Kuwait City, KWT; 2 Dermatology, Kuwait Institute for Medical Specializations, Kuwait City, KWT; 3 Dermatology, Musaed Al-Saleh Health Center, Kuwait City, KWT

**Keywords:** apremilast, biologic therapy, psoriasis, psoriatic arthritis, secukinumab

## Abstract

Psoriasis is a chronic, immune-mediated inflammatory skin condition with variable clinical courses and therapeutic responses. We present the case of a 55-year-old female with chronic plaque psoriasis refractory to multiple biologic agents, including conventional systemic immunosuppressants, Janus kinase inhibitors, and interleukin inhibitors. Despite sequential biologic regimens, the patient demonstrated only partial and transient improvement. Given the persistence of severe disease, combination therapy with secukinumab, an IL-17A inhibitor, and apremilast, a phosphodiesterase-4 inhibitor, was initiated. This dual approach resulted in significant clinical improvement and eventual disease resolution, with sustained remission over follow-up. The case highlights the potential role of combination targeted therapy in managing biologic-refractory psoriasis and emphasizes the need for individualized treatment strategies in complex or resistant cases.

## Introduction

Psoriasis is a chronic, immune-mediated inflammatory disorder characterized by erythematous, scaly plaques resulting from keratinocyte hyperproliferation and dysregulated immune responses. It affects approximately 2%-3% of the global population, with the latest systematic review estimating a prevalence of about 4.4% worldwide and regionally showing Asia at ~ 5.7% [1]. The disease thus poses substantial physical, psychological, and socioeconomic burdens, including impairment in health-related quality of life and increased disability adjusted life years (DALYs) [2]. Increasing evidence supports its classification as a systemic disease, frequently associated with comorbidities such as psoriatic arthritis, metabolic syndrome, cardiovascular disease, and depression. For instance, one recent meta-analysis estimated that the prevalence of psoriatic arthritis among patients with psoriasis is around 17.6% [3].

The pathogenesis of psoriasis arises from a complex interplay of genetic, environmental, and immune factors. Activation of Th1 and Th17 pathways, with cytokines such as TNF-α, IL-17, and IL-23, drives epidermal hyperplasia and chronic inflammation. Biologic therapies targeting these pathways have revolutionized management; however, some patients experience incomplete or diminishing responses, necessitating combination or adjunctive treatment strategies. Among these, secukinumab, an IL-17A inhibitor, and apremilast, a phosphodiesterase-4 (PDE-4) inhibitor, offer complementary mechanisms, blocking pro-inflammatory cytokine release through distinct immune pathways [4]. Clinical experience suggests that, although monotherapy with each agent has demonstrated robust efficacy, the evidence for their combined use remains limited to case reports and small series. For example, one case report documented successful treatment of highly refractory plaque psoriasis with the combination of apremilast and secukinumab [5].

Here, we present a case of chronic plaque psoriasis that was refractory to multiple lines of biologic therapy. The patient subsequently achieved significant and sustained clinical improvement following the introduction of a combined regimen of secukinumab and apremilast. This case highlights the potential therapeutic benefit of combination biologic and small-molecule therapy in managing difficult-to-treat psoriasis and contributes to the growing body of evidence supporting individualized treatment strategies for refractory disease.

## Case presentation

A 55-year-old Iraqi female presented with a five-year history of pruritic, scaly plaques involving the palms, soles, knees, shins, elbows, forearms, and scalp. The lesions were gradually progressive and associated with intermittent itching and discomfort. The disease followed a chronic, relapsing course without complete remission.

**Figure 1:**
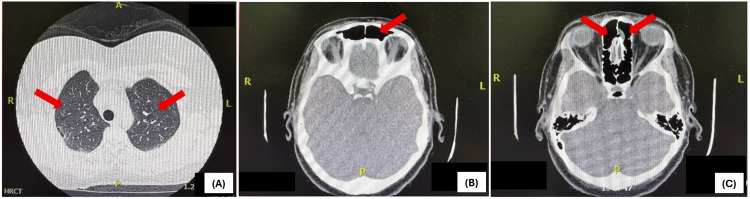
Imaging Findings From Past Medical History (A) HRCT showing bilateral emphysematous changes consistent with COPD. (B) Paranasal sinus imaging demonstrating mild left frontal sinusitis. (C) Paranasal sinus imaging demonstrating mild bilateral ethmoid sinusitis. HRCT: High-Resolution Computed Tomography; COPD: Chronic Obstructive Pulmonary Disease

Her past medical history was notable for chronic plaque psoriasis with psoriatic arthritis. She also had multiple comorbidities, including chronic obstructive pulmonary disease (COPD), with high-resolution computed tomography (HRCT) revealing bilateral emphysematous changes; obstructive sleep apnea, managed with continuous positive airway pressure (CPAP) (Figure 1a); and ischemic heart disease, status post coronary angiography showing mild mid-left anterior descending (LAD) artery bridging, currently managed medically. Additional comorbidities included dyslipidemia, fibromyalgia on duloxetine, and hypothyroidism on levothyroxine therapy. The patient was a chronic active smoker with a 35-year history. Gastrointestinal evaluation revealed gastroesophageal reflux disease (grade A), a large (grade IV) hiatal hernia, gastritis, and duodenitis. Paranasal sinus imaging demonstrated mild bilateral ethmoid and left frontal sinusitis (Figure 1b).

**Figure 2:**
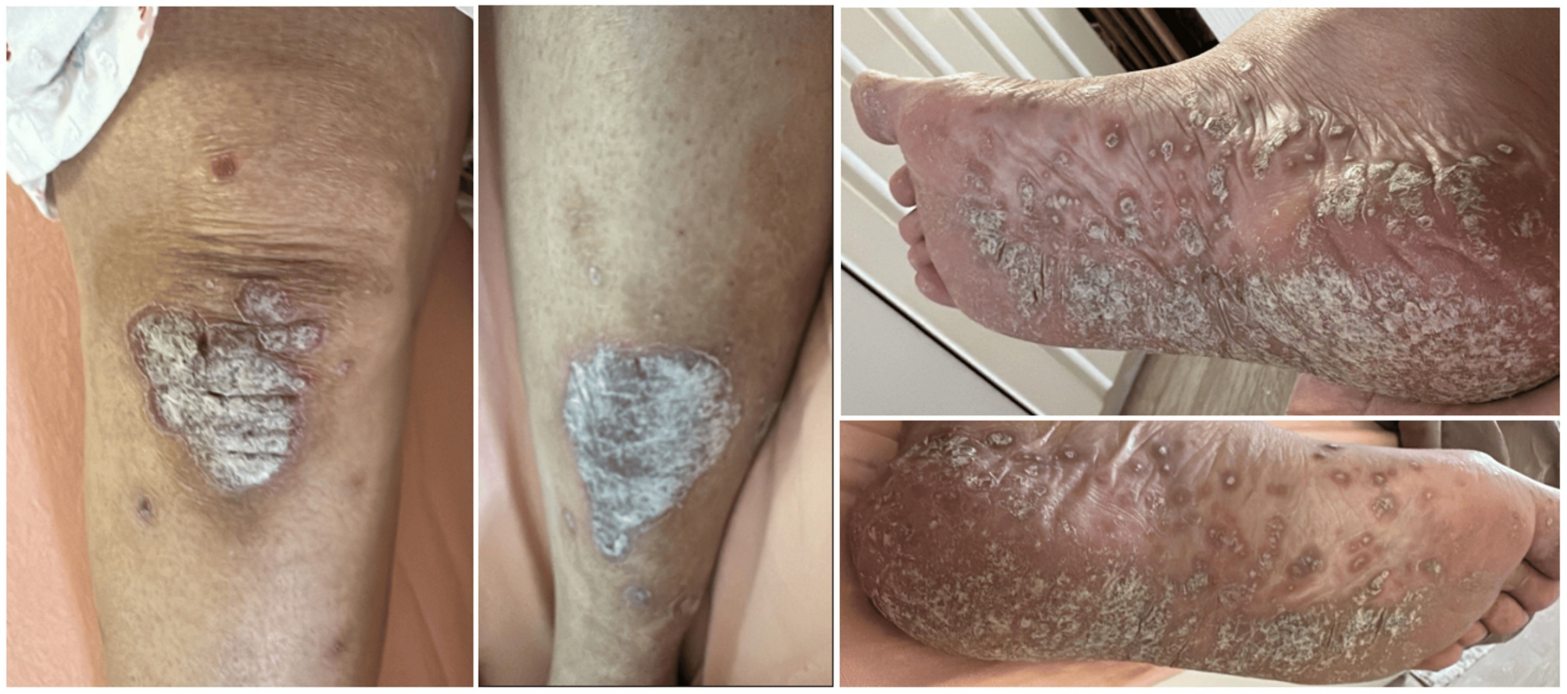
Clinical Presentation of Chronic Plaque Psoriasis Affecting the Lower Extremities Well-defined, erythematous plaques with thick, adherent silvery-white scales symmetrically distributed over the knees, shins, and plantar surfaces.

General physical examination revealed a middle-aged female in no acute distress, with stable vital signs. Cutaneous examination showed multiple, well-defined, erythematous plaques with thick, adherent, silvery-white scales distributed symmetrically over the palms, soles, knees, shins, elbows, forearms, and scalp. The plaques varied in size from a few centimeters to confluent areas, with surrounding mild erythema and occasional fissuring over the palms and soles. The lesions were pruritic, chronic, and recurrent, with no evidence of pustulation, exudation, or secondary infection. Palmar and plantar surfaces exhibited hyperkeratotic, fissured plaques, causing mild discomfort on movement. Extensor surfaces such as the elbows and knees were prominently involved, with well-circumscribed plaques demonstrating classic psoriatic scaling (Figure 2).

**Figure 3:**
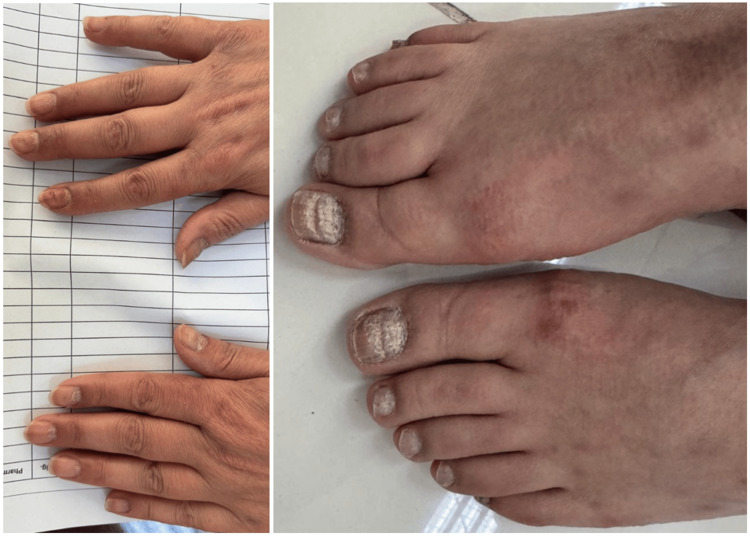
Psoriatic Nail Changes in Fingernails and Toenails Psoriatic nail changes demonstrating typical features, including pitting, onycholysis, subungual hyperkeratosis, and longitudinal ridging, with greater severity in the toenails compared to the fingernails.

The scalp showed diffuse scaling and erythematous plaques extending slightly beyond the hairline. Nail examination revealed changes characteristic of psoriasis, including nail pitting, onycholysis, subungual hyperkeratosis, and longitudinal ridging, more prominent in the toenails than fingernails (Figure 3).

Mucous membranes were normal with no oral or genital involvement. Musculoskeletal examination demonstrated tenderness, swelling, and limited range of motion in the distal and proximal interphalangeal joints of both hands, consistent with psoriatic arthritis. There were no deformities or dactylitis noted at the time of examination. Systemic examination, including respiratory, cardiovascular, and gastrointestinal systems, was unremarkable apart from findings related to her known comorbidities.

The patient was initially managed by the rheumatology service for active psoriatic arthritis and chronic plaque psoriasis. The diagnosis was made clinically, based on characteristic, well-demarcated erythematous, scaly plaques and nail changes, without the need for skin biopsy.

Baseline laboratory investigations, including complete blood count, renal and liver function tests, lipid profile, and inflammatory markers (ESR and CRP), were performed prior to initiating systemic therapy (Table 1). Screening for hepatitis B and hepatitis C was negative (Table 2). Screening for latent tuberculosis was performed using an interferon-gamma release assay (IGRA) and was negative. Baseline laboratory tests were within normal limits and remained stable throughout treatment. The patient was up to date on routine and influenza vaccinations prior to initiating biologic therapy. Periodic clinical surveillance for *Candida *infection during IL-17 inhibitor therapy revealed no mucocutaneous or systemic involvement. No adverse events were reported, including weight loss, gastrointestinal intolerance, or mood disturbances.

**Table 1:** Baseline Laboratory Investigations Prior to Initiation of Systemic Therapy ALT: alanine transaminase; AST: aspartate aminotransferase; CRP: C-reactive protein; ESR: erythrocyte sedimentation rate

Test	Value	Reference range (Unit)
WBC	7.6	4-10 (10^9^/L)
RBC	4.6	3.8-4.8 (10^12^/L)
Haemoglobin	136	120-150 (g/L)
Platelet count	370	150-410 (10^9^/L)
Lymphocytes	2.7	1-3 (10^9^/L)
Creatinine	58	49-90 (umol/L)
Sodium	138	136-144 (mmol/L)
Potassium	4.5	3.6-5.1 (mmol/L)
Total protein	78	66-83 (g/L)
Albumin	44	35-52 (g/L)
Total bilirubin	6.6	5-21 (umol/L)
ALT	20	3-35 (U/L)
AST	17	3-35 (U/L)
Total cholesterol	6	3-5.2 (mmol/L)
Triglyceride	1.94	0.4-1.75 (mmol/L)
CRP	9	0-8 (mg/L)
ESR	30	0-20 (mm/hr)

**Table 2:** Baseline Screening for Hepatitis B and Hepatitis C Prior to Systemic Therapy

Virology	
Test	Results
HBs Ag in blood	Non-reactive
Anti HCV in blood	Non-reactive

**Figure 4:**
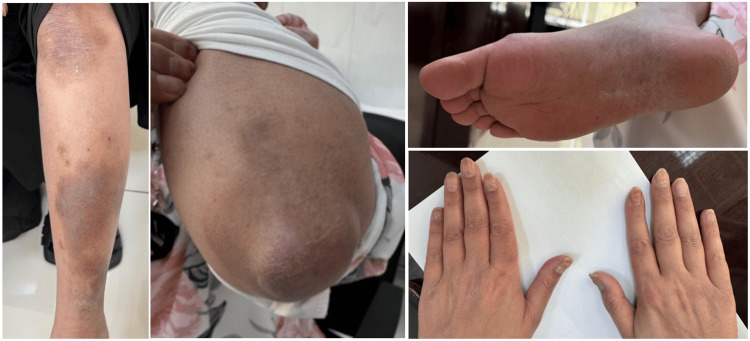
Resolution of Psoriatic Lesions and Nail Changes Following Combination Therapy Marked resolution of erythematous plaques and scaling is observed after six months of treatment, with mild residual post-inflammatory hyperpigmentation on the lower extremities, plantar surfaces, and elbows, and significant improvement in nail changes.

The patient was started on ixekizumab (Taltz) 80 mg subcutaneously once monthly. Despite good adherence, no significant improvement in either joint or skin symptoms was observed during follow-up visits. Her regimen was subsequently changed to guselkumab (Tremfya) 100 mg subcutaneously at weeks 0 and four, followed by maintenance dosing every eight weeks. After eight weeks of therapy, the patient continued to exhibit active psoriatic arthritis and persistent psoriatic plaques, prompting the addition of methotrexate (MTX) 25 mg subcutaneously once weekly to the Tremfya regimen. In the absence of satisfactory clinical response, Tremfya was discontinued, and the patient was reinitiated on Taltz 80 mg subcutaneously once monthly, again in combination with MTX 25 mg subcutaneously weekly. However, no notable improvement was achieved. Subsequently, upadacitinib (Rinvoq) 15 mg orally once daily was introduced alongside MTX 25 mg subcutaneously weekly, but the disease remained active. Given the refractory nature of her condition, she was referred to the combined dermatology-rheumatology clinic for multidisciplinary assessment. After evaluation, the dermatologist initiated secukinumab (Cosentyx) 300 mg subcutaneously weekly for five weeks, followed by 300 mg every two weeks, in combination with apremilast (Otezla) 30 mg orally twice daily (Table 3). Following this regimen, the patient demonstrated marked improvement in cutaneous lesions, with significant reduction in erythema, scaling, and pruritus (Figure 4).

**Table 3:** Chronologic Therapeutic Timeline and Clinical Response Chronologic overview of systemic and biologic therapies showing treatment duration, dosing, and response. The patient demonstrated marked improvement and sustained remission only after initiating combination therapy with secukinumab (IL-17A inhibitor) and apremilast (PDE-4 inhibitor). Note: All transitions between therapies included standard washout intervals (four to eight weeks depending on half-life and clinical activity) and no overlapping biologics.

Year/Duration	Therapy	Dose & Schedule	Treatment Duration	Response	Reason for Switch/Notes
2020-2021	Ixekizumab (Taltz)	80 mg SC every 4 weeks	8 months	No significant skin or joint improvement	Primary non-response
2021-2022	Guselkumab (Tremfya)	100 mg SC at week 0, 4 → q8w	6 months	Partial improvement	Inadequate response
2022-2023	Guselkumab + Methotrexate	100 mg q8w + MTX 25 mg SC weekly	6 months	Minimal improvement	Persistent active disease
2023	Ixekizumab + Methotrexate	80 mg monthly + MTX 25 mg weekly	4 months	No improvement	Secondary inefficacy
2023-2024	Upadacitinib + Methotrexate	15 mg PO daily + MTX 25 mg weekly	5 months	No clinical benefit	Refractory psoriatic arthritis
2024-Present	Secukinumab + Apremilast	300 mg SC weekly × 5 → q2w + Apremilast 30 mg BID	Ongoing > 12 months	Marked cutaneous and joint improvement within 3 months	Sustained remission; well-tolerated

Additionally, the severity of psoriatic arthritis decreased, with improvement in joint pain and stiffness, although mild residual arthritic activity persisted. Lifestyle modification, including weight management, regular exercise, and smoking cessation, was also strongly advised as part of the comprehensive management plan.

Before initiating combination therapy, the patient had severe, uncontrolled psoriasis with PASI 18, BSA 15%, and DLQI 22, along with active psoriatic arthritis. Despite multiple biologic and systemic regimens, skin and joint symptoms remained active with minimal improvement. Six months after starting secukinumab and apremilast, the patient achieved PASI 90 with residual post-inflammatory hyperpigmentation and partial nail improvement. Response was maintained at 12 months with no new lesions or flares. Psoriatic arthritis improved to an ACR50 response and disease activity in psoriatic arthritis (DAPSA) low disease activity, with stable laboratory results and no adverse events or infections.

## Discussion

Psoriasis is a chronic, immune-mediated inflammatory skin disease characterized by hyperproliferation and abnormal differentiation of keratinocytes, driven by dysregulated interactions between T cells, dendritic cells, and cytokines, such as TNF-α, IL-17, and IL-23. It affects approximately 2-3% of the global population and follows a chronic, relapsing-remitting course [6].

Several clinical variants of psoriasis are recognized, with chronic plaque psoriasis being the most common, accounting for nearly 80-90% of cases. Other forms include guttate psoriasis, which presents as a sudden eruption of multiple small, round or oval, erythematous papules with fine scaling typically following streptococcal infection; inverse psoriasis, involving intertriginous areas; pustular psoriasis, which may be localized or generalized; erythrodermic psoriasis, a severe inflammatory variant involving most of the body surface, results in skin failure and an inability to maintain essential homeostatic functions; and nail psoriasis, which may occur alone or in association with cutaneous lesions [7].

Plaque psoriasis typically presents as well-demarcated, salmon-pink plaques covered with loosely adherent silvery-white scales, most commonly affecting the extensor surfaces, such as the elbows, knees, scalp, and lumbosacral region. The lesions often appear symmetrically and may be accompanied by variable degrees of pruritus. Nail involvement - including pitting, onycholysis, oil spots, subungual hyperkeratosis, and dystrophy - occurs in up to half of patients and is frequently associated with psoriatic arthritis. Psoriatic arthritis, an inflammatory arthropathy affecting approximately 20-30% of individuals with psoriasis, may involve the distal interphalangeal joints, axial skeleton, or entheses, resulting in pain, stiffness, and progressive deformity if left untreated [8].

Beyond joint and skin manifestations, psoriasis is increasingly recognized as a systemic inflammatory disease associated with multiple components of the metabolic syndrome, including hypertension, dyslipidemia, obesity, and insulin resistance - all of which contribute to an increased risk of cardiovascular morbidity and mortality. Furthermore, psoriasis has been linked to inflammatory bowel disease and a higher incidence of certain malignancies, such as lung, colorectal, and renal cancers, particularly among patients who smoke or are obese. From a psychosocial standpoint, the visible and often uncomfortable nature of psoriatic lesions can lead to depression, anxiety, and social withdrawal, exacerbated by stigma and misconceptions surrounding skin diseases. Collectively, these findings underscore the multifaceted nature of psoriasis as a chronic, systemic condition that demands comprehensive and multidisciplinary management [9].

Psoriasis is a chronic, immune-mediated inflammatory disorder characterized by dysregulation of the innate and adaptive immune systems, leading to keratinocyte hyperproliferation, aberrant epidermal differentiation, acanthosis, neovascularization, and potent cutaneous infiltration by immune cells. The disease arises from a complex interplay of genetic predisposition, immune dysfunction, and environmental triggers [10].

At the molecular level, activation of dendritic cells and T lymphocytes, particularly Th1, Th17, and Th22 subsets, drives the release of key proinflammatory cytokines, including tumor necrosis factor-alpha (TNF-α), interleukin (IL)-17, IL-23, and IL-22. These cytokines stimulate keratinocytes to proliferate and secrete additional inflammatory mediators, creating a self-amplifying inflammatory loop within the skin. The IL-23/IL-17 axis is now recognized as central to psoriasis pathogenesis and serves as the target for several biologic therapies, such as secukinumab and ixekizumab [4].

Genetic factors also play a significant role, with strong associations identified at the PSORS1 locus on chromosome 6p21, particularly involving the HLA-C*06:02 allele. These genetic variants contribute to altered immune signalling and enhanced susceptibility to disease development and chronicity [11].

Several environmental and lifestyle factors can trigger or exacerbate psoriasis in genetically predisposed individuals. These include infection (especially streptococcal pharyngitis), stress, skin trauma (Koebner phenomenon), certain medications (e.g., beta-blockers, lithium, and antimalarials), and climate changes (e.g., air pollutants and sun exposure). Moreover, smoking, obesity, and alcohol consumption are well-established risk factors that not only increase disease severity but also contribute to treatment resistance and associated comorbidities [11].

The diagnosis of psoriasis is primarily clinical, relying on the characteristic morphology and distribution of lesions. Plaque psoriasis, the most common form, typically presents with well-demarcated, salmon pink plaques covered by loosely adherent silvery-white scales, most often affecting the extensor surfaces, scalp, and lumbosacral region. Classical diagnostic signs such as the Auspitz sign, manifesting as pinpoint bleeding upon removal of scales, and the Koebner phenomenon, in which lesions develop at sites of skin trauma, further support the clinical diagnosis [12].

In the present case, the diagnosis was made clinically at the bedside, based on the typical distribution of lesions over the palms, soles, elbows, knees, shins, forearms, and scalp, in addition to nail involvement characterized by pitting, onycholysis, and subungual hyperkeratosis. The presence of concomitant psoriatic arthritis reinforced the diagnosis, and no biopsy was required. While histopathology is not routinely necessary, it can be valuable in atypical cases or when differential diagnoses need to be excluded. Typical histologic features of psoriasis include hyperkeratosis, regular acanthosis with elongated rete ridges, parakeratosis, loss of the granular cell layer, leukocyte infiltration, and dilated tortuous capillaries in the dermal papillae. Two hallmark histopathological features of psoriasis are the presence of Munro microabscesses, which are focal collections of neutrophils within the parakeratotic stratum corneum, and spongiform pustules of Kogoj, characterized by neutrophilic aggregates within the spinous layer of the epidermis [13].

Laboratory and imaging investigations are generally aimed at evaluating systemic involvement, monitoring for comorbidities, and ensuring safe treatment selection. Baseline blood tests, including complete blood count, liver and renal function tests, are important for patients receiving systemic therapies such as methotrexate or biologics. Inflammatory markers such as erythrocyte sedimentation rate and C-reactive protein may be elevated in cases with active psoriatic arthritis. Assessment of metabolic syndrome components, including lipid profile, fasting glucose, and HbA1c, is relevant due to the strong association of psoriasis with cardiovascular risk factors. Imaging studies, such as X-rays or MRI of affected joints, can provide valuable information on the extent and severity of psoriatic arthritis, revealing erosions, joint space narrowing, or, in advanced cases, the classic “pencil-in-cup” deformity [14].

Dermoscopy plays a valuable role in the non-invasive evaluation and monitoring of psoriasis. It provides a horizontal view of the skin, revealing the characteristic vascular patterns of psoriatic plaques - uniformly distributed dotted, pinpoint, or coiled (glomerular) vessels on a light red background with white diffuse scales. These vascular structures correspond to dilated capillaries within elongated dermal papillae, and their identification helps distinguish psoriasis from other inflammatory dermatoses such as lichen planus or pityriasis rubra pilaris [15].

The differential diagnosis of psoriasis varies with clinical presentation. Classic plaque psoriasis is usually straightforward to recognize; however, atypical cases and variant forms can mimic other dermatoses, making accurate diagnosis more challenging. Common conditions that may resemble plaque psoriasis include atopic dermatitis, nummular eczema, lichen simplex chronicus, pityriasis rosea, pityriasis rubra pilaris, and tinea infections. Scalp psoriasis must be distinguished from seborrheic dermatitis or tinea capitis, while palmoplantar lesions may resemble lichen planus, hand dermatitis, or contact dermatitis. Pustular psoriasis, whether localized or generalized, can mimic dyshidrotic eczema, folliculitis, allergic contact dermatitis, Stevens-Johnson syndrome, toxic epidermal necrolysis, or acute generalized exanthematous pustulosis. Guttate psoriasis may be confused with pityriasis rosea, pityriasis lichenoides, lymphomatoid papulosis, tinea versicolor, or secondary syphilis, whereas erythrodermic psoriasis requires differentiation from drug reactions, atopic dermatitis, congenital ichthyoses, bullous dermatoses, cutaneous T-cell lymphoma, and pityriasis rubra pilaris. Inverse psoriasis can resemble intertrigo, fungal or bacterial infections, Hailey-Hailey disease, Darier disease, extramammary Paget disease, or other intertriginous disorders [14].

Nail findings in psoriasis, including pitting, onycholysis, oil spots, and subungual hyperkeratosis, also have a broad differential and may overlap with conditions such as alopecia areata, contact dermatitis, systemic lupus erythematosus, onychomycosis, or trauma. In uncertain cases, histopathology, mycological testing, or other laboratory evaluations can help confirm the diagnosis and distinguish psoriasis from its mimics [12].

The assessment of psoriasis severity is a critical component in guiding treatment decisions and evaluating therapeutic response. Several standardized scoring systems have been developed to quantify disease extent and activity, the most widely used being the Psoriasis Area and Severity Index (PASI). The PASI combines the evaluation of erythema, induration, and scaling across four body regions - head, trunk, upper limbs, and lower limbs - each weighted by the percentage of body surface area (BSA) involved. Scores range from 0 to 72, with higher scores reflecting more severe disease. Typically, mild psoriasis is defined by a PASI score below 10 and BSA involvement of less than 10%, while moderate to severe disease corresponds to PASI ≥10 or BSA ≥10%. In clinical practice, the BSA and the Dermatology Life Quality Index (DLQI) are also routinely used alongside PASI. BSA provides a rapid estimate of disease extent, while DLQI measures the psychosocial burden of psoriasis, recognizing the profound impact on quality of life. A DLQI score greater than 10 generally indicates significant impairment warranting systemic therapy [16].

The management of psoriasis is highly individualized, guided by disease severity, extent of BSA involvement, impact on quality of life, and presence of psoriatic arthritis or other systemic comorbidities. Treatment strategies are generally categorized into topical, phototherapy, systemic, and biologic modalities, often used sequentially or in combination to achieve optimal control [11].

For mild-to-moderate psoriasis, first-line therapy typically involves topical agents, including corticosteroids, vitamin D analogues (calcipotriol), calcineurin inhibitors, and keratolytic preparations. The efficacy of topical treatment can be increased with occlusion or combination therapy. These target localized plaques by reducing keratinocyte proliferation and inflammation. However, in moderate-to-severe or refractory disease, systemic and biologic therapies become essential [8].

Conventional systemic agents such as methotrexate, cyclosporine, and acitretin remain widely used due to their cost-effectiveness and established efficacy. Nonetheless, their long-term use is often constrained by hepatotoxicity, nephrotoxicity, or teratogenicity, necessitating laboratory monitoring and treatment rotation [11].

Biologic therapies represent the last-line yet most efficacious treatment option for psoriasis, offering targeted suppression of the immune-mediated inflammatory processes that drive disease activity in autoimmune disorders. Depending on the specific pathway inhibited, a wide range of biologic agents are available for the management of moderate-to-severe psoriasis. The advent of these therapies has revolutionized disease management by focusing on key immune mediators, such as TNF-α, IL-12/23, IL-17, and IL-23. Agents including adalimumab, ustekinumab, and secukinumab have demonstrated superior efficacy and safety compared with traditional systemic treatments. Nevertheless, a subset of patients may experience partial or diminishing responses over time, often due to factors such as immunogenicity, subtherapeutic serum levels, or heterogeneity in cytokine pathway dominance [13].

In such refractory cases, combination or sequential biologic therapy may be considered, although this approach remains uncommon and requires careful supervision. Although both ixekizumab and secukinumab target IL-17A, they differ in molecular composition, pharmacokinetic exposure, and immunogenicity, which can lead to differential efficacy among nonresponders. Limited data suggest that intra-class switching or dose optimization can restore response in patients with inadequate outcomes to an initial IL-17A inhibitor. In our case, secukinumab was reintroduced at an intensified schedule (300 mg weekly × 5, then every two weeks), achieving a meaningful clinical response. Apremilast, a phosphodiesterase-4 inhibitor, exerts anti-inflammatory effects by modulating intracellular cyclic AMP and downregulating pro-inflammatory cytokines. The decision to add apremilast rather than transition to another biologic class was guided by its distinct mechanism of action, which complements IL-17 blockade without overlapping immunosuppression. Apremilast’s oral administration, favorable safety profile, and evidence of synergistic efficacy when combined with biologics supported its selection as an adjunct in this refractory case. This dual-targeted approach resulted in marked improvement of cutaneous lesions and reduction in psoriatic arthritis severity, highlighting the potential synergy between targeted biologic and small-molecule pathways in difficult-to-treat psoriasis. Our findings align with previous observations by De et al. [17], who reported two patients with psoriasis achieving substantial improvement and dose reduction of secukinumab when co-administered with apremilast, without additional adverse effects [17]. Our observations are also supported by the systematic review by Arora et al. [18], which recommended combining newer biologics, including IL-17 inhibitors, with small-molecule agents such as apremilast to optimize response and maintain safety in psoriatic disease. Treatment optimization may also involve dose escalation or interval shortening of biologics in partial responders, provided safety and tolerance are maintained. Continuous monitoring for infection, metabolic comorbidities, and psychological well-being remains essential for holistic management [18].

The prognosis of psoriasis varies based on disease severity, comorbidities, and therapeutic response. In patients with mild disease, effective topical therapy often allows long-term control, and quality of life can be preserved. However, moderate-to-severe psoriasis - especially when associated with psoriatic arthritis, metabolic syndrome, or cardiovascular comorbidities - carries a more guarded prognosis due to the risk of systemic complications. Early initiation of systemic or biologic therapies, adherence to treatment, and management of comorbid conditions are critical for achieving sustained remission and preventing irreversible joint damage or organ dysfunction. Despite these interventions, psoriasis remains a chronic relapsing disorder, and many patients experience intermittent flares. Long-term outcomes are generally favorable with modern biologic and targeted therapies, particularly when therapy is tailored to individual immune profiles and disease patterns, although vigilance is required for potential adverse effects and secondary treatment failure [19].

## Conclusions

This case highlights the therapeutic potential of combining secukinumab (IL-17A inhibitor) and apremilast (PDE-4 inhibitor) in managing refractory chronic plaque psoriasis with psoriatic arthritis. The patient achieved and maintained PASI 90 and DAPSA low disease activity over a 12-month follow-up period without adverse effects. While these findings suggest a promising and well-tolerated dual-target approach, confirmation of its efficacy and safety requires validation in larger cohorts and long-term studies.
